# Variations in pulsatile flow around stenosed microchannel depending on viscosity

**DOI:** 10.1371/journal.pone.0210993

**Published:** 2019-01-24

**Authors:** Hyeonji Hong, Jae Min Song, Eunseop Yeom

**Affiliations:** 1 School of Mechanical Engineering, Pusan National University, Busan, South Korea; 2 Department of Oral and Maxillofacial Surgery, School of Dentistry, Pusan National University, Busan, South Korea; Universidade de Lisboa Instituto Superior Tecnico, PORTUGAL

## Abstract

In studying blood flow in the vessels, the characteristics of non-Newtonian fluid are important, considering the role of viscosity in rheology. Stenosis, which is an abnormal narrowing of the vessel, has an influence on flow behavior. Therefore, analysis of blood flow in stenosed vessels is essential. However, most of them exist as simulation outcomes. In this study, non-Newtonian fluid was observed in stenosed microchannels under the pulsatile flow condition. A polydimethylsiloxane channel with 60% stenosis was fabricated by combining an optic fiber and a petri dish, resembling a mold. Three types of samples were prepared by changing the concentrations of xanthan gum, which induces a shear thinning effect (phosphate buffered saline (PBS) solution as the Newtonian fluid and two non-Newtonian fluids mimicking normal blood and highly viscous blood analog). The viscosity of the samples was measured using a Y-shaped microfluidic viscometer. Thereafter, velocity profiles were analyzed under the pulsatile flow condition using the micro-particle image velocimetry (PIV) method. For the Newtonian fluid, the streamline was skewed more to the wall of the channel. The velocity profile of the non-Newtonian fluid was generally blunter than that of the Newtonian fluid. A highly oscillating wall shear stress (WSS) during the pulsatile phase may be attributed to such a bluntness of flow under the same wall shear rate condition with the Newtonian fluid. In addition, a highly viscous flow contributes to the variation in the WSS after passing through the stenosed structures. A similar tendency was observed in simulation results. Such a variation in the WSS was associated with plaque instability or rupture and damage of the tissue layer. These results, related to the influence on the damage to the endothelium or stenotic lesion, may help clinicians understand relevant mechanisms.

## 1. Introduction

Since the characteristics of pulsatile blood flow in the vessels are complex and unsteady, it is not easy to comprehend hemodynamic features completely.[1, 2] To understand blood flow, studies using Newtonian fluids, such as mixtures of glycerol and water models, have been widely conducted.[3–8] The results on Newtonian fluids may be reasonable for large-scale channels mimicking arteries. However, the non-Newtonian behavior of the blood increases the viscosity at a low shear strain rate.[9, 10] Blood viscosity influences flow resistance, and an increased viscosity is a biological parameter related to cardiovascular disease.[11] Therefore, Newtonian blood flow may insufficiently illustrate actual cardiovascular flow at low shear rate regions, such as downstream of stenosis in small-diameter vessels.

Stenosis, formed by deposition of cholesterol and other relevant lipids, abnormally narrows the blood vessels affecting flow behavior.[12] As the blood flows through stenosed channels, hemodynamic characteristics, such as velocity vector, wall shear rate (WSR), and wall shear stress (WSS), calculated by multiplying blood viscosity and WSR are changed.[13] Low and high WSS distributions involve the cap strength and determine not only the plaque rupture location but also the timing.[14–16] In simulation studies, it was reported that vessel structures are associated with repetitive flow phases and temporal variations in the WSS.[17] Considering the important role of the WSS in the formation and progression of atherosclerosis, it is necessary to examine pulsatile blood flow around the stenosed vessel.

Non-Newtonian flow under the pulsatile condition was utilized in studies; however, most analyses were reported using simulation results.[2, 12, 18–23] Many have conducted numerical studies to analyze the distributions of parameters around stenotic structures, such as velocity, WSS, and pressure.[12, 18, 20–24] For example, Amornsamankul et al. showed noticeable effects of non-Newtonian pulsatile flow on the velocity profile and WSS in their numerical study.[22] Tian et al. simulated a pulsatile blood flow focusing on the geometrical parameters of stenosis.[20] Nandakumar et al. compared numerical data such as WSS and velocity between Newtonian and shear-thinning fluids in aorta-size model.[24] Rabby et al. more concretely reported different fluctuations in pressure and the WSS in Newtonian and non-Newtonian fluids under the pulsatile flow condition.[19] Walker et al. conducted an experiment on blood flow patterns under the pulsatile condition.[25] Nevertheless, investigations under such conditions are more insufficient in terms of micro-scale vessels. Vahidkhah et al. simulated red blood cell (RBC) flow in microchannels with and without stenosis; however, the inlet flow condition was not a pulsatile flow but a steady flow.[26] Kumar et al. compared steady and pulsatile flows between blood and water in various microchannels without stenosis.[27] Conversely, previous experimental studies have analyzed flow velocity profiles using the micro-particle image velocimetry (PIV) method. Lima et al. and Yeom et al. used RBC suspensions in a non-circular microchannel.[28, 29] Actually, RBCs can be considered as a tracer particle in PIV measurement. However, it is adequate to apply an artificial tracer particle rather than RBCs, since measurement using RBCs can underestimate data for high magnification using the micro-PIV method.[30] It can result from a relatively bigger size or nonuniform distribution of RBCs (approximately 8 μm in diameter). Non-Newtonian fluids, such as xanthan gum solution, have been utilized as a blood-mimicking fluid in several experiments to measure the flow distribution accurately.[25, 31, 32]

In this study, pulsatile flow in a polydimethylsiloxane (PDMS) microchannel with stenosis was measured using the micro-PIV method with respect to the non-Newtonian behavior of samples. The velocity profiles and WSS were analyzed depending on the phase states and the positions in the microchannel. To measure the value of the WSS accurately, viscosity was also evaluated depending on the shear rates using a Y-shaped microfluidic viscometer.[33] Velocity fields and 3D distribution of WSS were monitored through a numerical simulation.

## 2. Materials and methods

### 2.1. Fabrication of PDMS microchannel

**Fig 1:**
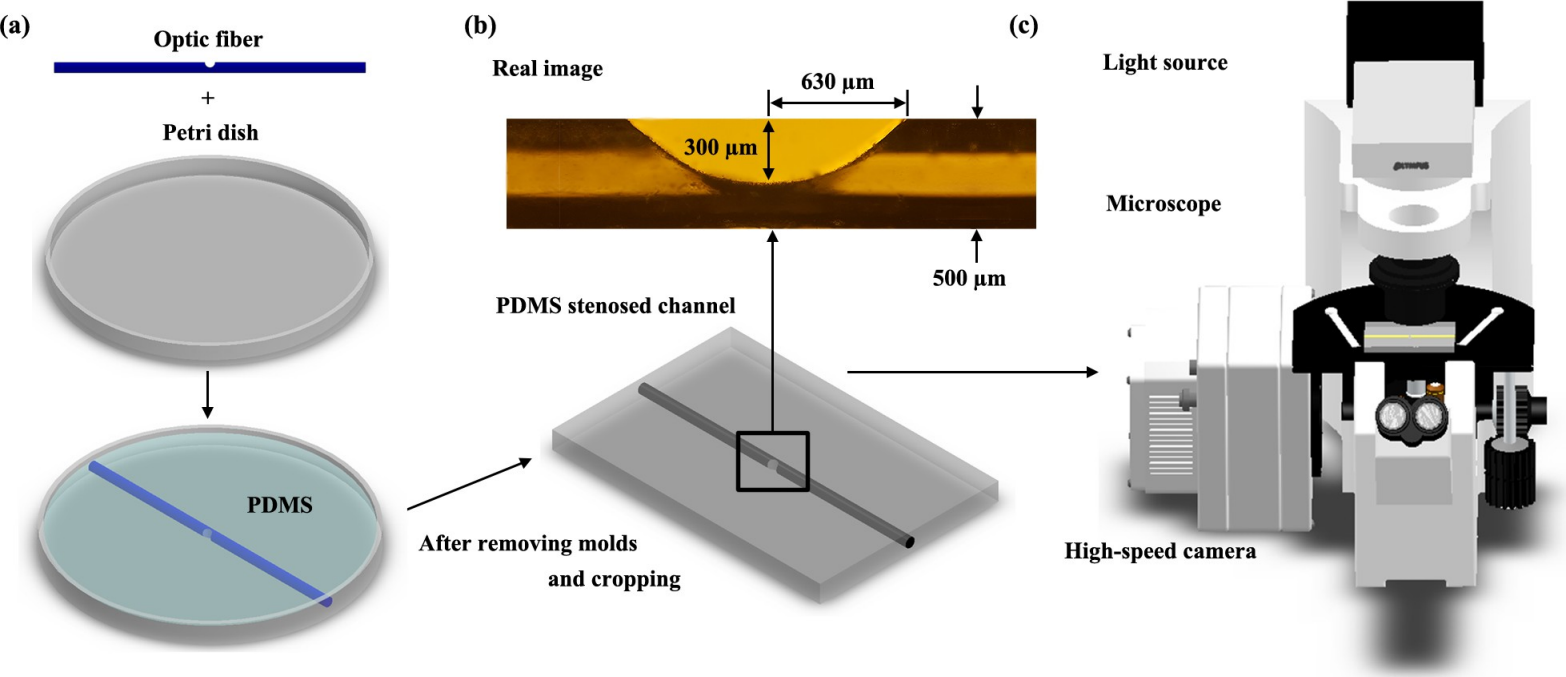
**(a)** Creation of the PDMS stenosed microchannel using a polymethylmethacrylate optic fiber and a petri dish. After combining the sanded optic fiber and petri dish, PDMS was poured into the mold and cured at 85°C. Finally, it was separated into a PDMS polymer, a dish, and an optic fiber; the PDMS stenosed channel was then cut into the desired shape. **(b)** Illustration of the fabricated PDMS stenosed channel (bottom) and actual image (top) captured using a microscope lens. The microchannel had a 500-μm diameter and 60% severity of stenosis. **(c)** The experimental setup comprised a light source, an optical microscope with an objective lens at 10× magnification (NA of 0.3), and a high-speed camera. The PDMS channel was mounted on the microscope and illuminated using a 100-W halogen lamp. PDMS, polydimethylsiloxane.

PDMS microchannel was fabricated using a polymethylmethacrylate optic fiber with a diameter of 500 μm.[34] To stimulate stenosis geometry in the microchannel, the optic fiber immobilized on a flat plate using a tape, except in the middle part, was sanded using a sharp edge of a sandpaper until its degree of stenosis reached 60% with the stenosis length of 1260 μm. Thereafter, the optic fiber was fixed in the center of a petri dish, and PDMS (Sylgard 184, Dow Corning, USA) was then poured into the mold (**Fig 1A**). It was completely cured at 85°C and then removed from the petri dish. And then, the optic fiber was pulled out from the PDMS channel. **Fig 1B** shows the schematic diagram of PDMS stenosed channel model and actual image captured through the microscope. By selecting the 60% severity of stenosis, the microchannel reflected the moderate stenosis state, which may progress depending on environmental influences.

### 2.2. Experimental setup

PDMS stenosed channel was mounted on an optical microscope (IX2, Olympus, Tokyo, Japan) with an objective lens at 10× magnification (NA of 0.3), as shown in **Fig 1C**. It was illuminated by a 100-W halogen lamp. Flows in the microfluidic device were consecutively captured using a high-speed camera (Phantom VEO710L, Vision Research Inc., NJ, USA) at 400 frames per second (fps). Samples were supplied into the microchannel using a programmable syringe pump (neMESYS, Centoni Gmbh, Germany) with a 1-mL plastic syringe (BD; Becton Dickinson, NJ, USA). All experiments were conducted at 25°C.

### 2.3. Working fluids

PBS solution was used as the Newtonian fluid sample. For the non-Newtonian fluid, two types of samples were prepared by mixing water, glycerol, and xanthan gum. Two non-Newtonian samples with different concentrations of xanthan gum, which is a polysaccharide, were created. Since xanthan gum has a shear thinning characteristic, the viscosity of xanthan gum solutions decreases with increased shear rates. **Fluid 1** consisted of 79.1% (v/v) distilled water, 20.9% (v/v) glycerol, and 0.21 g/L of xanthan gum for a blood analog fluid.[31] **Fluid 2** comprised 0.42 g/L of xanthan gum under the same volumetric ratio of water and glycerol. **Fluid 2** contained a two-fold higher concentration of xanthan gum than **Fluid 1** for observing the effect on flow characteristics under highly viscous blood analog.

### 2.4. Micro-PIV

For obtaining velocity information using the micro-PIV method, tracer particles were added in the sample fluids with 0.2% solid proportion. The particles were polymer microspheres with a 0.52-μm diameter (Fluoro-Max, Thermo SCIENTIFIC, USA). The center plane of 3-dimensional microchannel was focused with the appropriate depth by the optics of the imaging system. In the present setup, depth of correlation expressing the depth over which particles contribute to the cross-correlation analysis is about 20 μm.[29, 35, 36] After capturing images using a high-speed camera at 400 fps, they underwent post-processing, including cropping and masking. The sequence images were converted into velocity vector fields using a commercial program (PIVview2C, PIVTEC GmbH, Germany). The size of the interrogation window was 32 × 8 pixels with 50% overlapping. MATLAB software (Mathworks, USA) was utilized for analyzing velocity data.

### 2.5. Numerical simulation

The numerical solutions for the pulsatile flow in stenosed channel were determined using CFX 16.1 (ANSYS, Inc., USA). Channel modeling was designed by SolidWorks software (Dassault Systèmes SolidWorks Corp., USA) considering circular segment of stenosed part with circular radius of 811.5 μm and central angle of 101.85° (**S1 Fig**). The laminar regime of the momentum equation was applied. The conservation equations for the transient state were solved for the coupling of the velocity and pressure. In this analysis, a no-slip condition was applied to every wall of the channel. The convergence criteria were 10^−5^ for both the momentum and the continuity equations. To obtain equation for transient mass flow rate, the function of pulsatile flow was obtained by conducting FFT algorithm through the experimental data in MATLAB software. In **S2 Fig**, the mean velocity of inlet pulsatile flow was represented with respect to time. The obtained function of mass flow rate was applied at the inlet of the channel. To minimize the influence of initial flow conditions, all simulations were carried out for three cycles (total time of 30s with time step of 0.1s). The inlet channel was extended to 5D length to make fully developed flow. An open condition with a relative pressure of 0 Pa was employed at the outlet of the channel.

The measured viscosity curves of non-Newtonian fluids were fitted by the Carreau-Yasuda model equation (**Table 1**).[37] The fitting parameters were taken in each case of **Fluid 1** and **Fluid 2**. **S3 Fig** represents dynamic viscosity of three samples at the maximum velocity phase (φ = 0.2). Non-Newtonian fluids including **Fluid 1** and **Fluid 2** have different viscosity distributions depending on the positions of stenotic geometry while Newtonian fluid, PBS, shows constant viscosity field. After checking with a preliminary grid dependency test, approximately 6,000,000 nodes were used in the present study.

**Table 1:** Constants for the Carreau-Yasuda model equation. *μ*_*∞*_ (viscosity at infinite shear rate), *μ*_*0*_ (viscosity at zero shear rate), *λ* (time constant), *a* (Yasuda exponent) and *n* (power law index).

	μ_∞_	μ_0_	λ	*a*	*n*
Case1	0.001894874	0.012062693	0.072938377	1.25	0.536498044
Case2	0.002307324	0.025894833	0.055079426	1.5	0.463126647

## 3. Measurement of the viscosity of the working fluids

**Fig 2:**
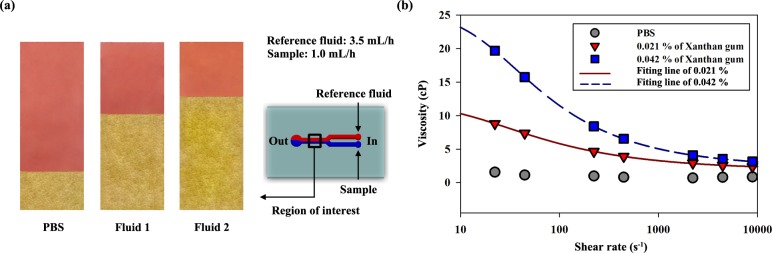
**(a)** Actual images captured using the optical microscope lens for phosphate buffered saline, **Fluid 1**, and **Fluid 2**. To identify the width ratios depending on the types of fluids, the flow rates were fixed at 3.5 mL/h for the reference fluid (labelled PBS) and 1.0 mL/h for the samples. The schematic diagram at the right side shows that the reference and sample fluids were injected respectively through each inlet and drained away from the outlet. The region of interest focused on the position where the two different fluids meet; the interface was then developed. **(b)** Viscosity variation depending on the shear rate of the three samples. The flow rate of the samples was changed from 0.05 to 20 mL/h.

To identify the viscosity of the three working fluid samples, a Y-shaped microchannel proposed in a previous study was used.[33] As shown in **Fig 2A**, the Y-shaped channel was composed of two inlets and one outlet. The downstream of the channel has a 3000-μm width and 50-μm height. The reference fluid and sample were delivered by syringe pumps into each inlet of the channel. The interfacial line was developed behind the junction of the Y-shaped microchannel where the two fluids meet. The flow image including an interfacial line (region of interest) was captured using a high-speed camera connected with the microscope. **Fig 2A** presents the actual images when the flow rates of the reference fluid and sample were fixed at 3.5 mL/h and 1.0 mL/h, respectively. The width ratio between the sample and reference fluid was determined by the pressure ratio determined by the flow rate and the viscosity of the fluids.

The viscosity of the sample was measured through pressure estimation based on the width ratio between the reference and sample flows. In other words, the viscosity information could be obtained by matching the relationship between the pressure and the width ratio for both flows.[33] To measure the precise width ratio, additional image processes were applied to the flow images. **Fig 2B** shows the measured viscosity depending on the shear rate of the three samples, i.e., PBS, **Fluid 1**, and **Fluid 2**. To adjust the shear rate of the sample flow, the flow rate was changed from 0.05 to 20 mL/h. PBS generally has a constant viscosity for a wide range of shear rate. As expected, the viscosity values of **Fluid 1** and **Fluid 2** decreased with the increase in the shear rate. The asymptotic viscosity became higher as the concentration of xanthan gum increased. Additionally, the shear thinning effect was also intensified. In order to describe the dynamic viscosity of the non-Newtonian solution (**Fluid 1** and **Fluid 2**), viscosity curve was fitted by the Carreau-Yasuda model equation;

$$\mu =\;\mu_{\infty }+\;\frac{\left(\mu_{0}-\mu_{\infty }\right)}{{\left(1+{\left(\lambda \dot{\gamma }\right)}^{a}\right)}^{\frac{1-n}{a}}}$$ (1)

here, μ_∞_, μ_0_, λ, *a* and *n* indicate the viscosity at infinite shear rate and zero shear rate, time constant, Yasuda exponent, and power law index, respectively (**Table 1**).

## 4. Results

### 4.1. Velocity variation in pulsatile flow

**Fig 3:**
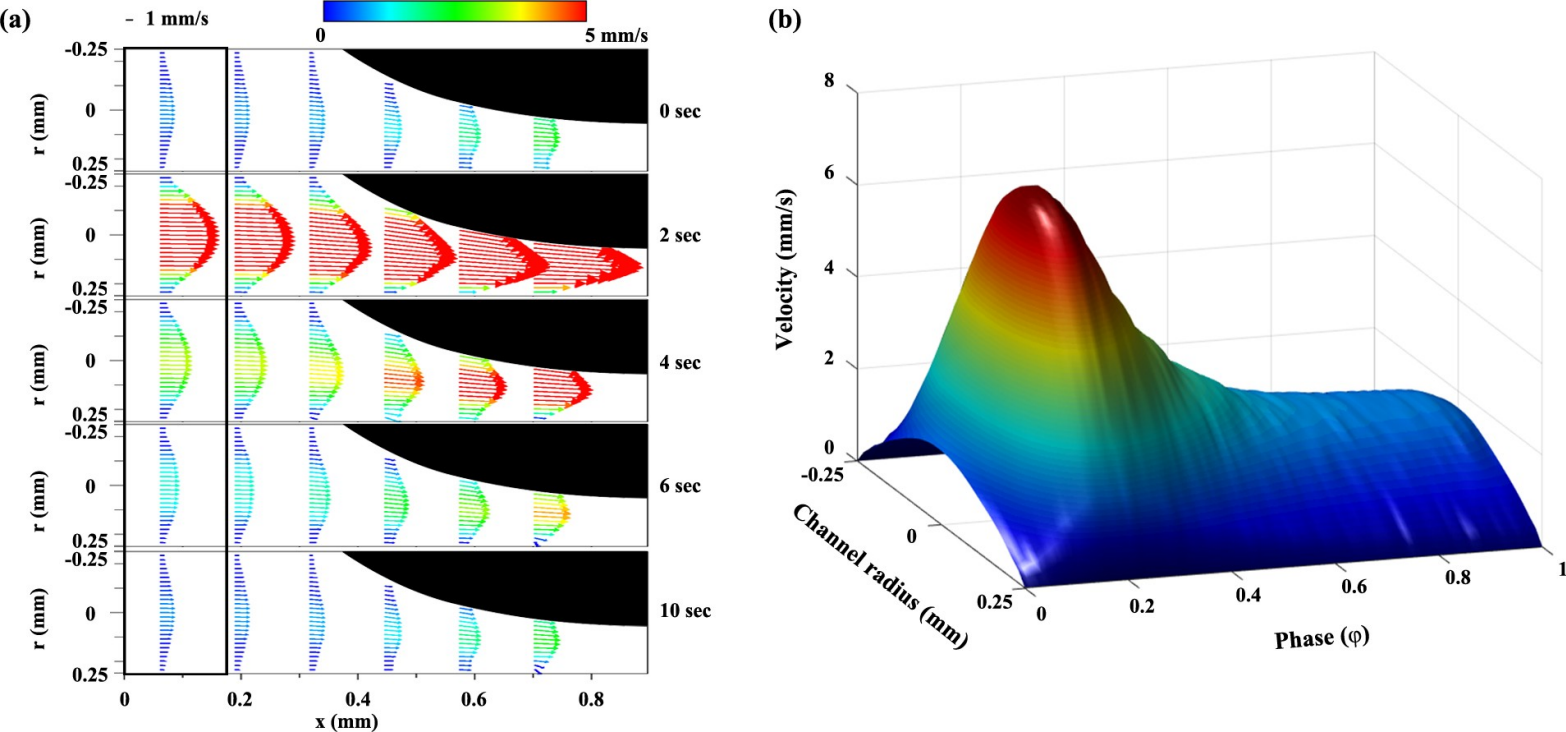
**(a)** Velocity vector fields of PBS at certain periods (0, 2, 4, 6, and 10 s) for pulsatile flow with 10-s periods per cycle. **(b)** Pulsatile inlet velocity distribution in a 3-dimensional plot, which was constructed using the velocity information in the bold black box (Fig 3A). Owing to the repetitive characteristic, the time variable was converted into phase (φ) states; thus, φ = 1 was the period per cycle.

The pulsatile flow was controlled based on the flow rate which is within 0.4–3 mL/h in all case. From that, Womersley number varies from 0.05 to 0.20 depending on the viscosities of samples (where Wo # = 0.5D(ωρ/μ)^0.5^). It denotes a dimensionless number of the pulsatile flow frequency in biofluid. **Fig 3A** shows the pulsatile inlet velocity distributions at 0, 2, 4, 6, and 10 s. The period of pulsatile flow was 10 s per cycle. The velocity was accelerated as the flow passed through the stenosed channel. The velocity profile in the black box was considered as the pulsatile inlet condition. **Fig 3B** depicts the inlet temporal variation in the velocity in 3-dimensions. Since the velocity profile repetitively varied owing to the pulsatile flow condition, the velocity was expressed depending on the phase (φ) states. Therefore, the period of one cycle was equal to φ = 1. The flow accelerated from φ = 0 to φ = 0.2, and the accelerated flow approached the maximum value at around φ = 0.2. The velocity steeply decreased in the early deceleration phase (from φ = 0.2 to φ = 0.4) and then gradually decreased until φ = 1.0.

**Fig 4:**
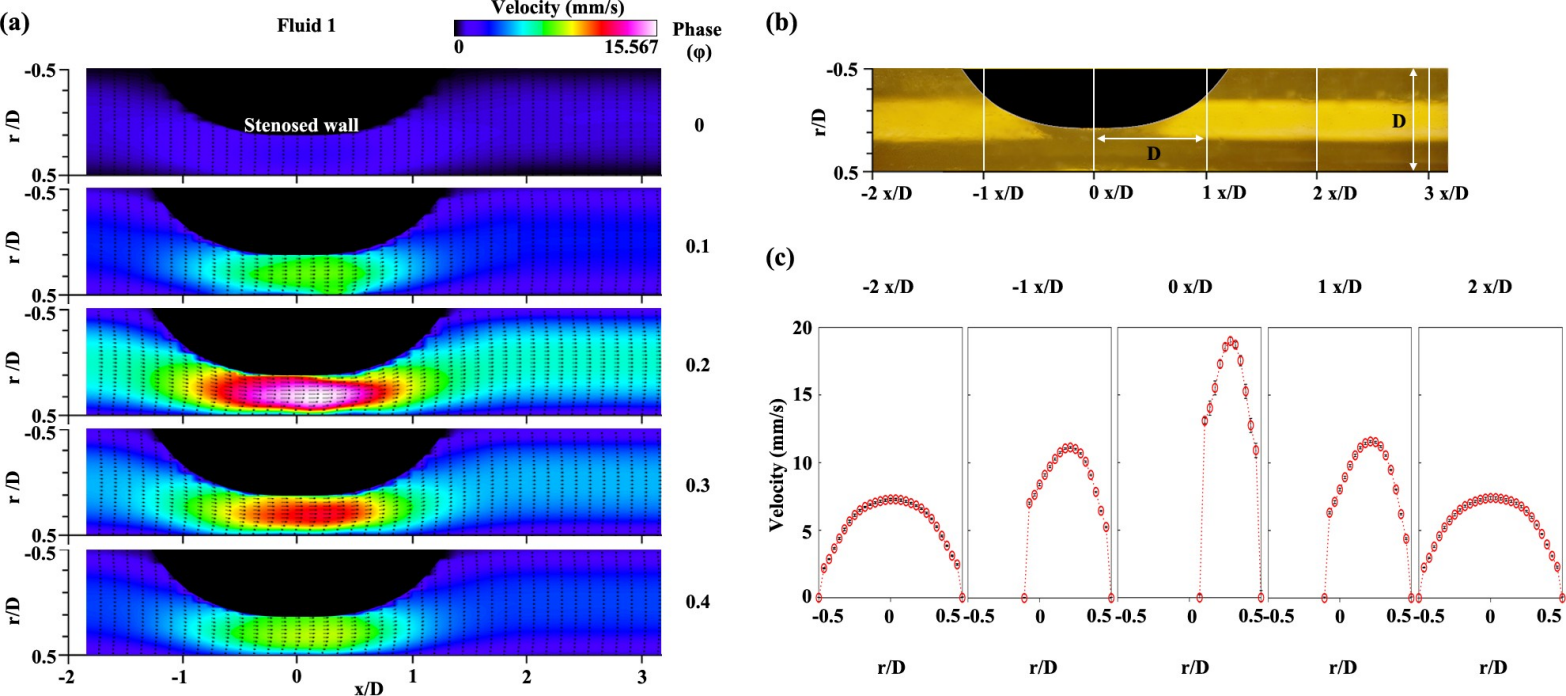
**(a)** Contoured velocity vector fields of **Fluid 1** at φ = 0, 0.1, 0.2, 0.3, and 0.4. The stenosed wall was at r/D = 0.1, and the opposite wall was at r/D = 0.5. **(b)** Definition of the x/D variable. D is the diameter of the channel, while x/D is the non-dimensional variable. The value of x/D was determined by the value of the x-coordinate, which was divided by the diameter of the channel when x = 0 was assigned at the center of the stenosis region in the x-coordinate. **(c)** Velocity profiles of **Fluid 1** extracted at a certain phase (φ = 0.2) depending on the positions (x/D = -2, -1, 0, 1, and 2). Each data set (mean ± standard deviation) is obtained from ensemble-average over 5 cycles.

**Fig 4A** shows the contoured velocity vector field for **Fluid 1** during acceleration and early deceleration phases from φ = 0 to φ = 0.4. In this channel, the stenosed wall was at r/D = 0.1, and the opposite wall was at r/D = 0.5, where D is the diameter of the microchannel used in the experiment. In all phase states, the velocity region at the stenotic apex accelerated. **Fig 4B** presents the positions at which the velocity was extracted for data analysis. x/D indicates a non-dimensional variable along the streamwise direction divided by the diameter of the channel. Thus, x/D = 0 was located at the center of the stenotic apex. In **Fig 4C**, the velocity profiles for **Fluid 1** were extracted at φ = 0.2 depending on the streamwise direction (x/D = -2, -1, 0, 1, or 2). As it passed through the stenosed channel, the maximum velocity in the profile value also varied from 7 mm/s to 20 mm/s. The absolute value of velocity at the stenosis region (x/D = 0) was two-fold higher than the value at the pre-stenosis region (x/D = -2).

### 4.2. Velocity vector fields for the Newtonian and non-Newtonian fluids

**Fig 5:**
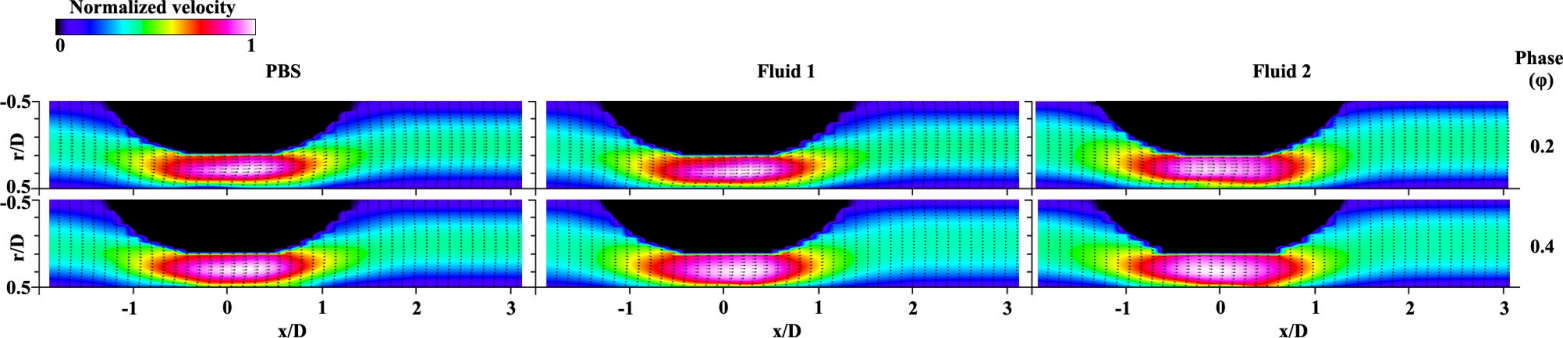
Contoured vector fields representing normalized velocity at φ = 0.2 and 0.4. In sequence from left to right, PBS, **Fluid 1**, and **Fluid 2** are classified.

**Fig 6:**
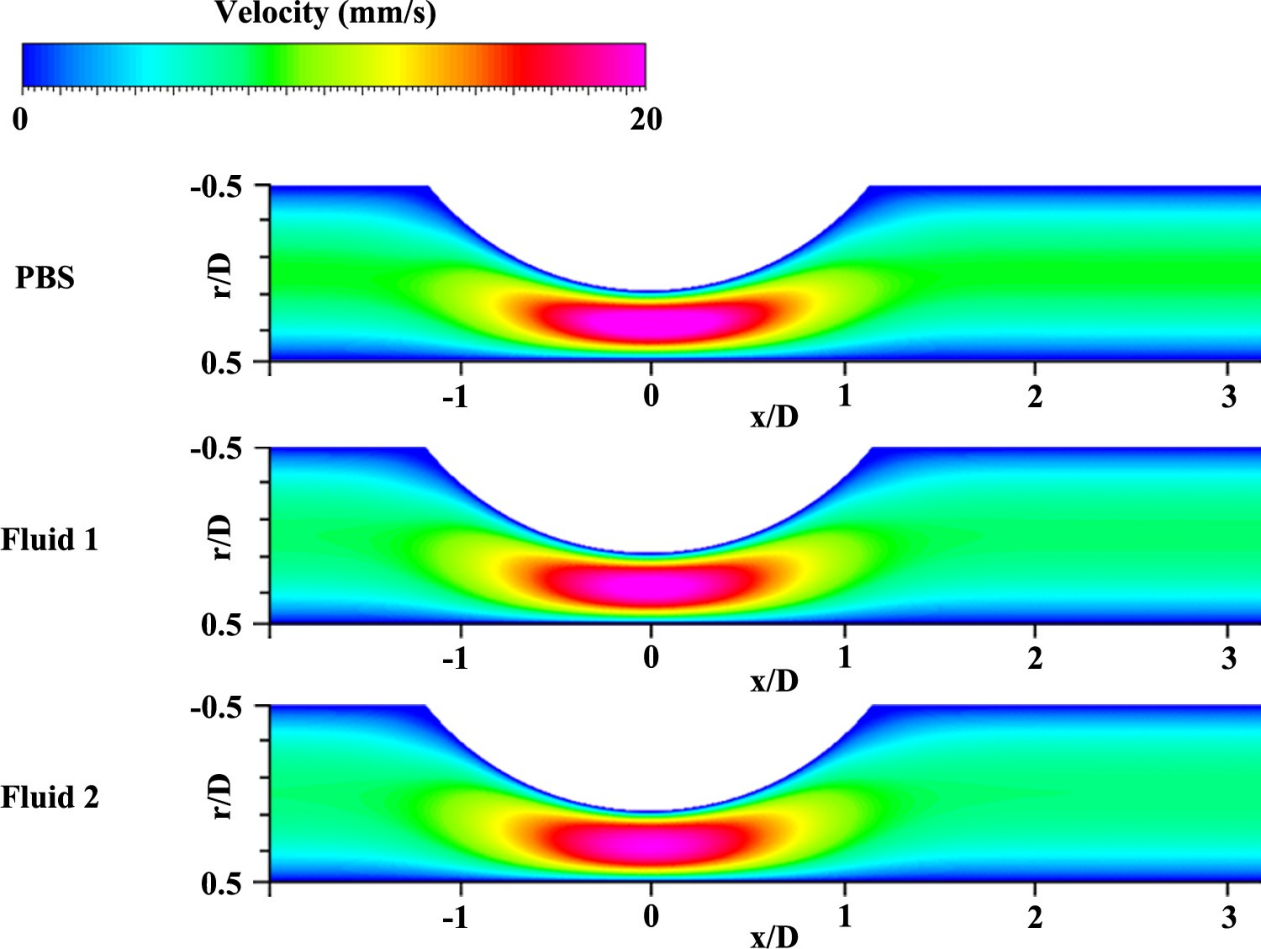
Simulation results representing contoured velocity fields at φ = 0.2 for PBS, Fluid 1, Fluid 2.

To compare the velocity distributions of the three samples, the contoured vector fields were arranged at φ = 0.2 and φ = 0.4, as shown in **Fig 5**. Each velocity field was normalized with its maximum velocity for easy comparison. The overall velocity distributions were similar in terms of the flow acceleration in front of the stenosis, maximum velocity region around the stenotic apex, and flow deceleration in the post-stenosis region. However, in the case of φ = 0.2, this high velocity region around the stenotic apex (around x/D = -1 to 1) was more skewed toward the stenosed wall (r/D = 0) than that in the case of φ = 0.4. At φ = 0.2, the inlet flow state was at the end of acceleration and then reached the maximum velocity. At the same time, it accelerated once again by sudden reduction of diameter, and the direction of the flow was then changed owing to the shape of the stenosis. Therefore, this streamwise flow tended to flow along the center of the longitudinal axis continuously and passed through the channel with high velocity, showing a skewed trend to the stenosed wall. The highly accelerated region during deceleration (φ = 0.4) appeared wider than that at φ = 0.2. Furthermore, the lowest velocity portion in **Fluid 1** and **Fluid 2** occupied the narrower region near the wall compared with that in PBS. It implies that the velocity profiles of **Fluid 1** and **Fluid 2** are blunter than the profile of PBS. **Fig 6** also shows velocity fields at φ = 0.2 from simulation results and it is similar with the trend of experimental results. For **Fluid 1** and **Fluid 2**, the velocity distributions are blunt since velocity region at the center of longitudinal axis has lower value and the lowest velocity region near the wall occupies narrower portion than the case of PBS.

### 4.3. Comparison of velocity profiles for Newtonian and non-Newtonian fluids

**Fig 7:**
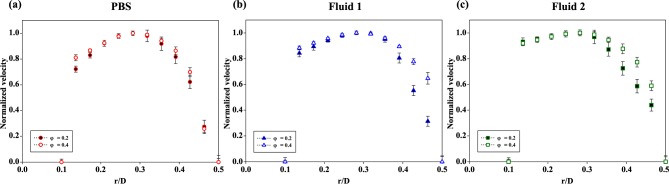
Normalized velocity profiles at the stenosis region (x/D = 0) of the three samples at φ = 0.2 and 0.4. The samples were **(a)** PBS, **(b) Fluid 1**, and **(c) Fluid 2**. The normalized diameter (r/D) indicates the stenosed wall side at r/D = 0.1 and the opposite wall side without stenosis at r/D = 0.5. Each data set (mean ± standard deviation) is obtained from ensemble-average over 5 cycles.

For quantitative analysis, the velocity profiles were extracted depending on the streamwise positions, phases, and types of the fluids. **Fig 7** shows the normalized velocity profiles of the three fluids at the stenotic apex (x/D = 0) for φ = 0.2 and φ = 0.4. The normalized diameter was within the range of r/D = 0.0–0.5; the convex wall at the stenosed side was situated on the center of the longitudinal axis at r/D = 0.1 and the opposite wall at the side without stenosis at r/D = 0.5. Owing to the asymmetrical stenosis shape, the distribution of the velocity profiles was skewed toward the stenosed wall. Moreover, the gradient of velocity near the stenosed wall was steep, since the stenotic structure interrupted the streamwise flow. Although these three profiles had similar trends, **Fluid 1** and **Fluid 2** tended to be blunter than PBS. In other words, the velocity profiles appear blunter as the viscosity of samples becomes higher irrespective of the phases. A high viscosity indicates that the friction between the molecules is also high; thus, the velocity values in r/D = 0.3–0.5 were flatter in **Fluid 2** than in **Fluid 1**. Considering the increased viscosity involving shear thinning in the deceleration phase (φ = 0.4), the bluntness was intensified when compared with the profile at the peak phase (φ = 0.2). Furthermore, the velocity difference between φ = 0.2 and φ = 0.4 in **Fluid 1** and **Fluid 2** was higher than that in PBS because the viscosity variation in the phase velocity intensified the difference in **Fluid 1** and **Fluid 2**.

**Fig 8:**
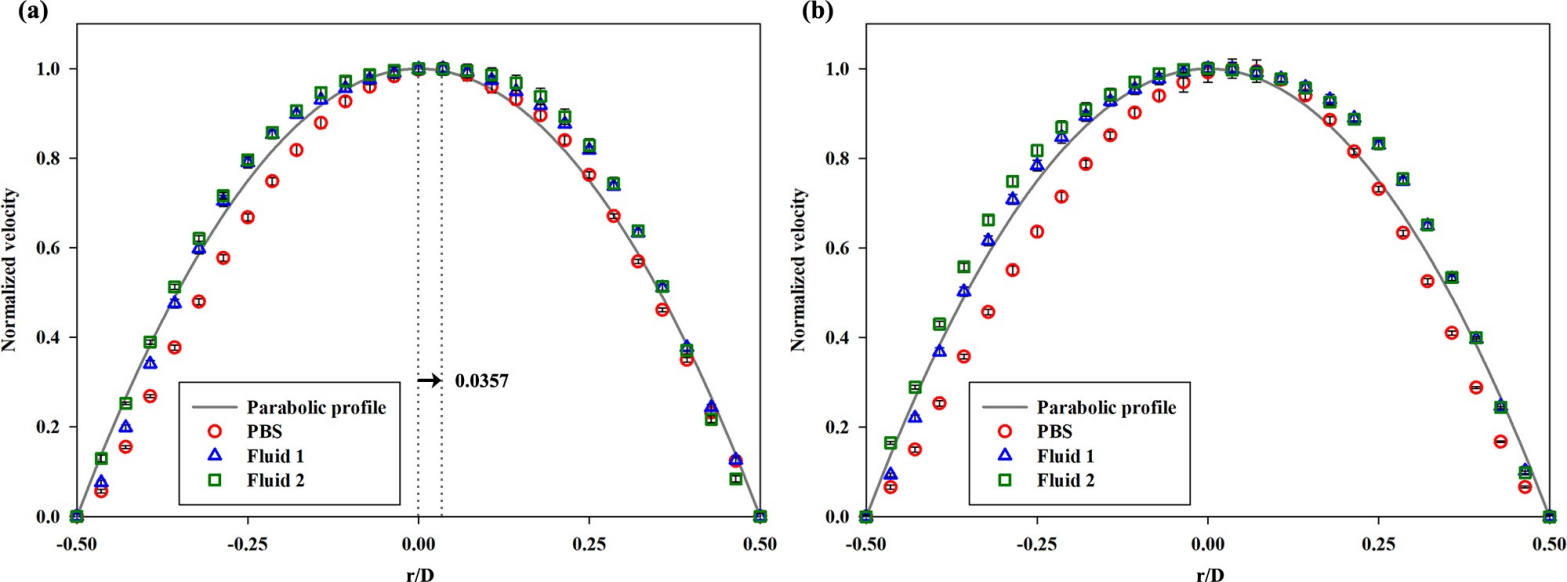
Normalized velocity profiles at the peak phase velocity (φ = 0.2) of PBS, Fluid 1, and Fluid 2. The points were extracted at the **(a)** pre-stenosis (x/D = -2) and **(b)** post-stenosis (x/D = 2) regions. As a reference, the parabolic profile is presented in a gray line. The value of 0.0357 in (a) indicates the position of the maximum value in the PBS profile. PBS, phosphate buffered saline. Each data set (mean ± standard deviation) is obtained from ensemble-average over 5 cycles.

**Fig 8** presents the normalized velocity profiles of the three different fluids at the pre-stenosis and post-stenosis regions at the phase of φ = 0.2 depending on the normalized diameter (r/D = -0.50–0.50). **Fig 8A** shows the upstream velocity profiles extracted at x/D = -2. To compare the shapes of the velocity profiles, a reference parabolic profile was inserted as a solid gray line. The profile of PBS was skewed from the center of the axis to the opposite wall (r/D = 0.50), while the other fluids had relatively symmetrical shapes. The high asymmetry of PBS (maximum velocity point; r/D = 0.0357) may have been observed because it is easy for Newtonian fluids to be affected by geometrical structures. The velocity profile of **Fluid 2** showed a blunter shape than those of the other fluids. **Fig 8B** indicates the downstream velocity profiles extracted at x/D = 2. The profiles of **Fluid 1** and **Fluid 2** were also slightly skewed towards the opposite wall of the stenosed wall after passing through the stenosis. From -0.50 to 0 in the normalized diameter, the difference in the values between **Fluid 1** and **Fluid 2** was intensified owing to the existence of an asymmetrical stenotic structure. Moreover, **Fluid 2** had the bluntest shape among the fluids at x/D = 2 owing to the highest viscosity. **Table 2**indicates the maximum velocity values of three samples and raw data of velocity profiles were provided by **S1 File**.

**Table 2:** Maximum velocity values (mm/s) of three samples at the stenosis apex (x/D = 0), pre-stenosis (x/D = -2) and post-stenosis (x/D = 2) for φ = 0.2 and 0.4.

	Phase (φ)	x/D = -2	x/D = 0	x/D = 2
PBS	0.2	7.11505	18.2598	7.40703
	0.4	4.16993	10.5137	4.30076
Fluid1	0.2	7.27285	18.4917	7.38645
	0.4	4.05417	10.4486	4.00911
Fluid2	0.2	6.72943	16.7716	6.64928
	0.4	3.97415	9.75317	3.89646

### 4.4. WSS distributions of the Newtonian and non-Newtonian fluids

Based on the measured velocity information for the three types of fluid in the microchannel, the WSR can be roughly estimated by employing the following simplified equation.

$$WSR\;=\;\frac{dv}{dr}\;\approx \;\frac{v\;(r_{w})-0}{r_{w}}$$ (2)

where v(r_w_) is the closest velocity data to the wall and r_w_ is the relevant distance from the wall in the r-coordinate. Considering the non-Newtonian feature of the samples (**S3 Fig**), the WSS can be reasonably calculated by multiplying the WSR obtained using Eq (2) and relevant viscosity at the corresponding WSR [μ (WSR)].

WSS=μ(WSR)⋅WSR (3)

**Fig 9:**
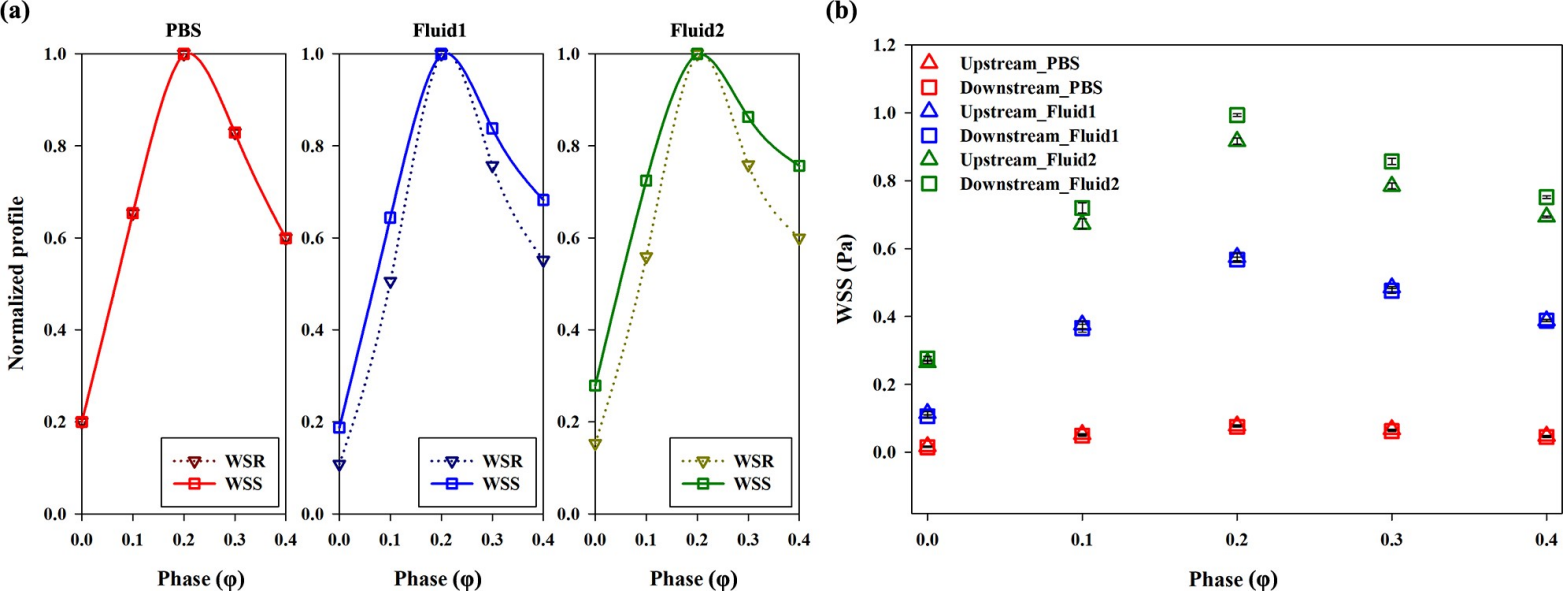
**(a)** Normalized profiles for the WSR and WSS depending on the phases (φ = 0.0–0.4), which are averaged for the stenosed wall and the opposite wall. PBS, **Fluid 1**, and **Fluid 2** in sequence from left to right. **(b)** WSS distributions in the three samples: PBS (red), **Fluid 1** (blue), and **Fluid 2** (green) at the pre- and post-stenosis regions (x/D = -2 and 2). Each data set (mean ± standard deviation) is obtained from ensemble-average over 5 cycles.

**Fig 9A** illustrates the changes in the WSR and WSS in PBS, **Fluid 1,** and **Fluid 2**. To compare the degree of variation among the parameters, each curve was normalized on the basis of their maximum value within the range of φ = 0.0–0.4. As expected, the Newtonian fluid had nearly the same WSR and WSS profiles. However, the non-Newtonian fluids showed dampened descending and ascending slopes for the WSS and a steeper slope for the corresponding WSR. In addition, this trend in **Fluid 2** was more intensified than that in **Fluid 1**, since the viscosities of these fluids increased with decreased shear rates; further, the degree of increased viscosity was higher in **Fluid 2** than in **Fluid 1**. **Table 3**presents the dimensional magnitudes of the WSR and WSS at φ = 0.0–0.4 for the three samples.

**Table 3:** Variations in WSR and WSS at the downstream of stenosis for three samples.

	Phase (φ)	0	0.1	0.2	0.3	0.4
PBS	WSR (s^-1^)	15.0716	49.2625	75.3585	62.4814	45.1620
	WSS (Pa)	0.0151	0.0493	0.0754	0.0625	0.0452
Fluid 1	WSR (s^-1^)	11.0218	51.6080	101.9915	77.2486	56.2654
	WSS (Pa)	0.1064	0.3658	0.5680	0.4761	0.3877
Fluid 2	WSR (s^-1^)	12.7454	46.5185	83.1970	63.1572	49.9137
	WSS (Pa)	0.2768	0.7199	0.9934	0.8571	0.7517

**Fig 10:**
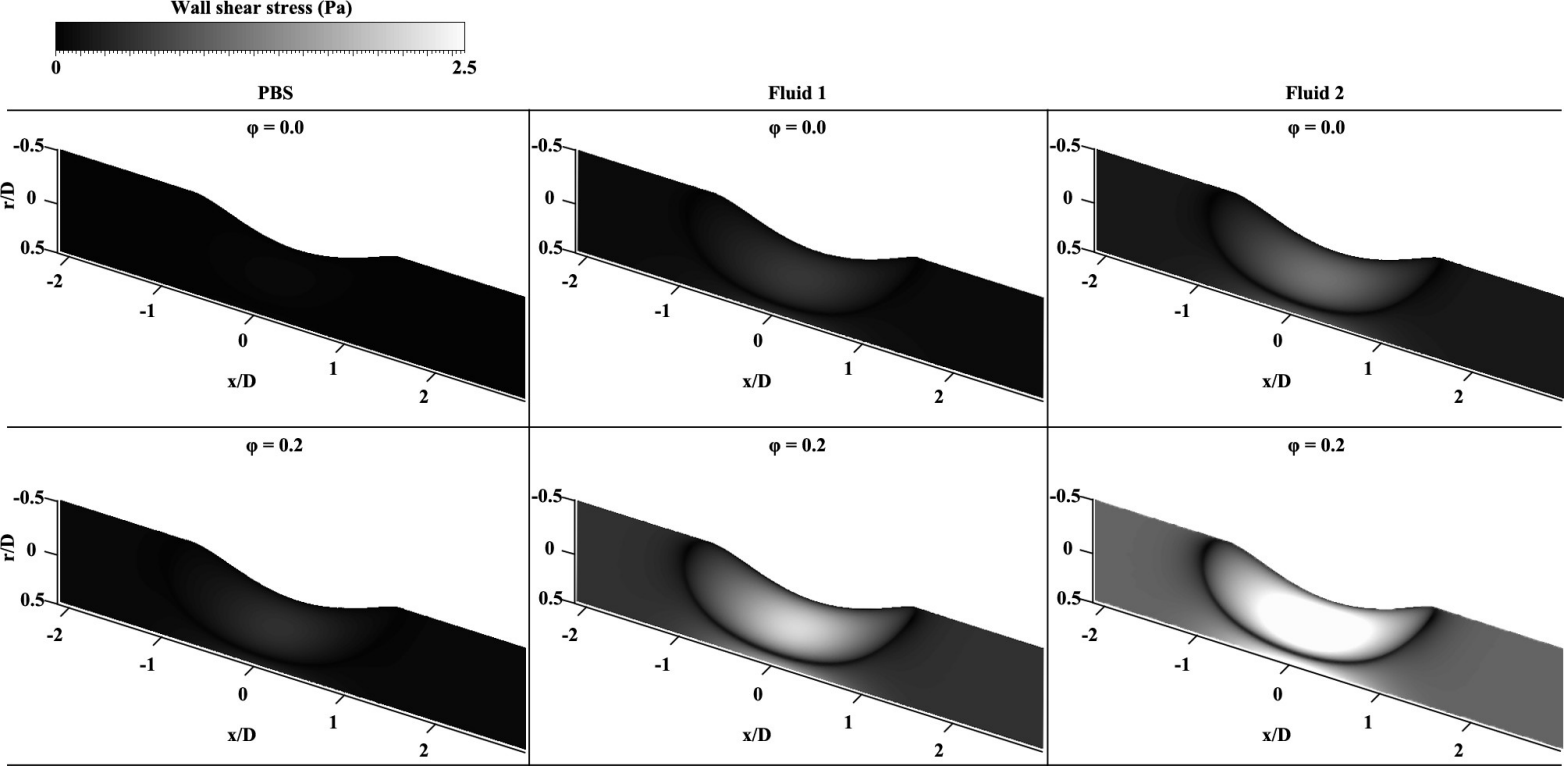
Simulation results representing WSS at φ = 0.0 and 0.2 in 3-demensional channel surface. In sequence from left to right, PBS, **Fluid 1**, and **Fluid 2** are classified.

**Fig 11:**
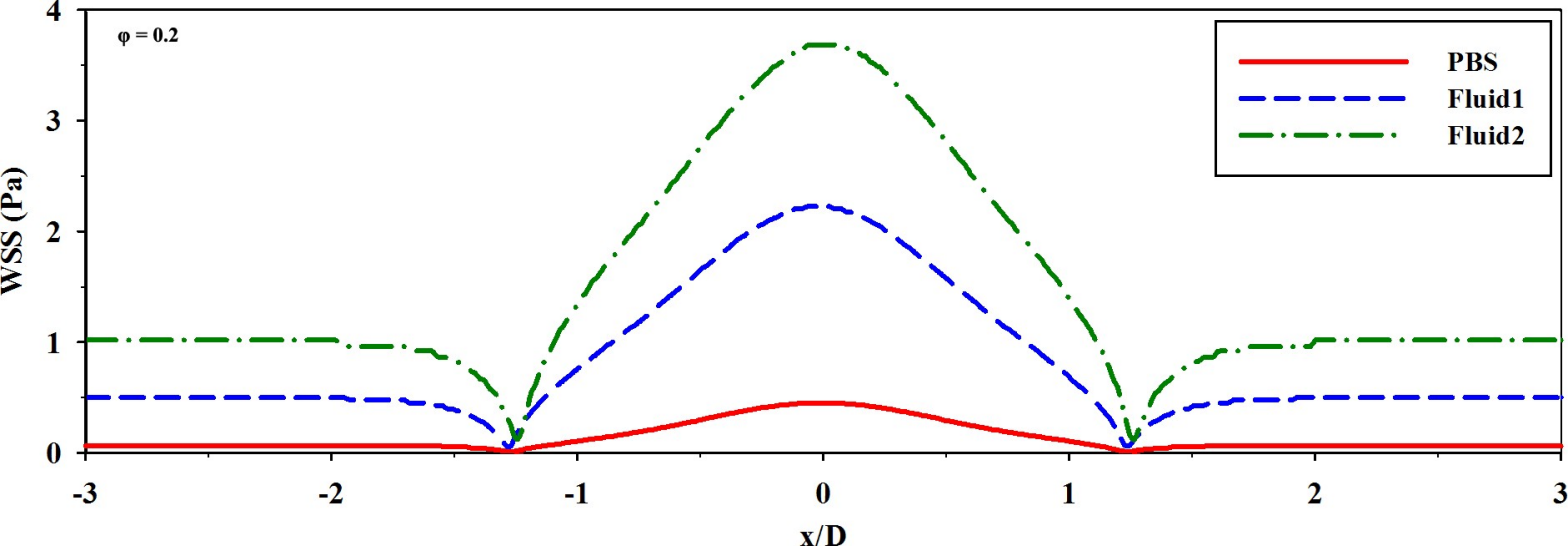
The distributions of the WSS for the stenosed wall along the flow direction according to the PBS, Fluid 1, and Fluid 2 (Simulation results).

**Fig 9B** shows the actual variations in the WSS at φ = 0.0–0.4 for the three fluids. To determine the variation induced by passing through the stenosed channel, each WSS distribution at the upstream and downstream of the stenosis (x/D = -2 and 2) was compared. For all samples, the WSS values increased in the acceleration regime (φ = 0.0–0.2) and decreased in the deceleration regime (φ = 0.2–0.4). At φ = 0.2, the WSS had a peak value, since the flow had the maximum velocity. For PBS, the variation in the WSS depending on the phase was not noticeable because the viscosity is constant and relatively low. The variations in the WSS in **Fluid 1** and **Fluid 2** were considerably higher than that in the Newtonian fluid. The variation tendency became more pronounced as the non-Newtonian fluids were accentuated. **Fluid 2** also showed the highest WSS value among the samples; this finding may be associated with the blunter velocity profile resulting from the higher viscosity. In **Fig 10**, WSS distributions of samples from the simulation results were represented at φ = 0.0 (diastolic phase) and 0.2 (systolic phase) in 3-dimension. In systolic phase, the WSS for the stenosed wall along the flow stream was compared depending on the samples (**Fig 11**). As the viscosity level of sample increased, the WSS around the stenosis also increased. It reflects similar trends in terms of the absolute magnitudes and the variations of WSS for three fluids, considering the experimental data. The high difference of WSS between upstream and downstream is observed in case of **Fluid 2**. Therefore, the increased viscosity can be considered as the influence factor on the high magnitude and variance of WSS as well as the distinct WSS at the pre-stenosis and the post-stenosis.

## 5. Discussion

The velocity profile of the Newtonian fluid was skewed toward the wall at the opposite side of the stenosed wall; such a skewness was not as intense in the non-Newtonian fluids. These findings imply that a Newtonian fluid can be easily skewed by geometric structures because its pressure loss is higher than that of a non-Newtonian fluid under the same flow condition in the curved geometry.[1, 12] Nevertheless, the shear thinning fluid was influenced by the stenosed structure. As shown in **Fig 8**, the velocity profile did not tend to be much skewed but seemed to be blunter at the post-stenosis region, since the pressure drop from upstream to downstream was smaller in the non-Newtonian model. Furthermore, the kinetic energy at the post-stenosis region drops owing to the effect of viscosity.[12, 25] From that, the bluntness is associated with the increase in blood viscosity, while the profile has a relatively high velocity at the vessel wall.[29] The velocity profile arising from the increased viscosity can alter the flow resistance because the frictional force can hinder RBCs from moving.[38, 39] Moreover, it may induce production of cholesterol and low-density lipoproteins.[11, 40] In **Fig 7**, the non-Newtonian fluids having a higher viscosity showed blunter shapes also at the stenotic apex than the Newtonian fluid. Therefore, for highly viscous fluids, a relatively high WSS around the stenosis region may be attributed to such a bluntness of flow under the same WSR condition with Newtonian fluids (**Fig 10**). A high WSS is associated with an increased risk of fibrous cap rupture.[41] Therefore, plaque remodeling may occur owing to a high WSS, which can result in acute coronary syndrome from rupture of the cap.[42–44]

Blood flow approaches Newtonian fluids at a high shear rate and non-Newtonian fluids at a low shear rate. Ashrafizaadeh and Bakhshaei demonstrated that the velocity profile of blood analog fluids approaches a more parabolic shape as the Reynolds number increases; further, that of Newtonian fluids assumes a different shape, despite the same geometry of the channel and flow rate conditions.[45] The WSR is related to the blunt shape of velocity profiles and shear layer for the shear thinning flow. [26] The flow characteristics result from the shear thinning nature at a low shear rate and influence the WSS and hemodynamic behaviors.[1, 31] Therefore, the results can be different in terms of velocity fields and shear stress between Newtonian and non-Newtonian fluids.[46] In addition, the nonlinearity of viscosity has an important role in the local flow and the flow change of pulsatile cycles.[47] Some studies have reported that the fluctuation in streamwise pressure occurs owing to stenosis in Newtonian fluids; conversely, the pressure gradually decreases along the channel in non-Newtonian fluids.[12, 21] The WSS also fluctuates less in non-Newtonian fluids; however, the magnitude is much higher at the stenosis region.[21] As mentioned above for the blood-mimicking fluid in **Fig 9**, the WSS had a dampened variation slope when compared with the relevant WSR variation. At the same time, high WSS magnitude occurred, and variance of WSS through the stenosed structure was apparent in the case of high viscosity fluid. The high magnitude of the WSS gradient contributes to endothelial cell alignment depending on the variation (positive or negative).[48] Furthermore, the combined effect between the WSS and WSS gradient is correlated with endothelial cell activation of relevant proteins.[49] The responses can result in vascular diseases, such as plaque instability or rupture and tissue layer damage.[50] Aimee et al. illustrated the various determinants of the blood viscosity, and suggested that the increased viscosity is an integral component of vascular disease.[51] Rainer et al. listed data showing the higher viscosity for patients with cardiovascular disease. Therefore, the complexity of comprehending hemodynamic features reiterates the importance of elucidating the variations in the parameters, such as velocity profiles and WSSs, in highly viscous fluids under pulsatile and geometrical conditions.

## 6. Conclusion

Through the experiment and simulation, the velocity profiles and WSS variations were observed in the stenosed microchannel along the phases of pulsatile flow using both Newtonian and non-Newtonian fluids. The blood analog flow can be affected by pulsatile flows and stenosed shapes. Such fluctuations may be related to potential heart attacks.[19, 25] Moreover, the increase in the WSS and the oscillating WSS in highly viscous fluids can damage the blood cells, endothelium, or stenotic lesions, since it is relevant to endothelial cell morphology and nitric oxide production.[25, 52–54] While this study cannot directly explain the mechanism related to vascular pathology, the lesion is associated with WSS and flow behavior caused by stenotic structure and pulsatile flow with regard to viscosity variations in blood analog fluids.

## Supporting information

S1 Fig Channel modeling with stenosed part (circular radius of 811.5 μm and central angle of 101.85°. (JPG)

S2 Fig Mean velocity profile for one cycle indicating inlet pulsatile flow condition applied to the numerical simulation. The mean velocity value is obtained by integrating 2D velocity profile of PBS for each time.
(JPG)

S3 Fig Dynamic viscosity distributions obtained by simulation results for PBS, Fluid 1, Fluid 2 at φ = 0.2. (JPG)

S1 File Raw data for Figs 2–4 and 7–9. (XLSX)
