# Supplementary figures and images for: Variations in pulsatile flow around stenosed microchannel depending on viscosity
Source: PLoS One. 2019 Jan 24;14(1):e0210993. doi: 10.1371/journal.pone.0210993 (PMC6345426; doi:10.1371/journal.pone.0210993)

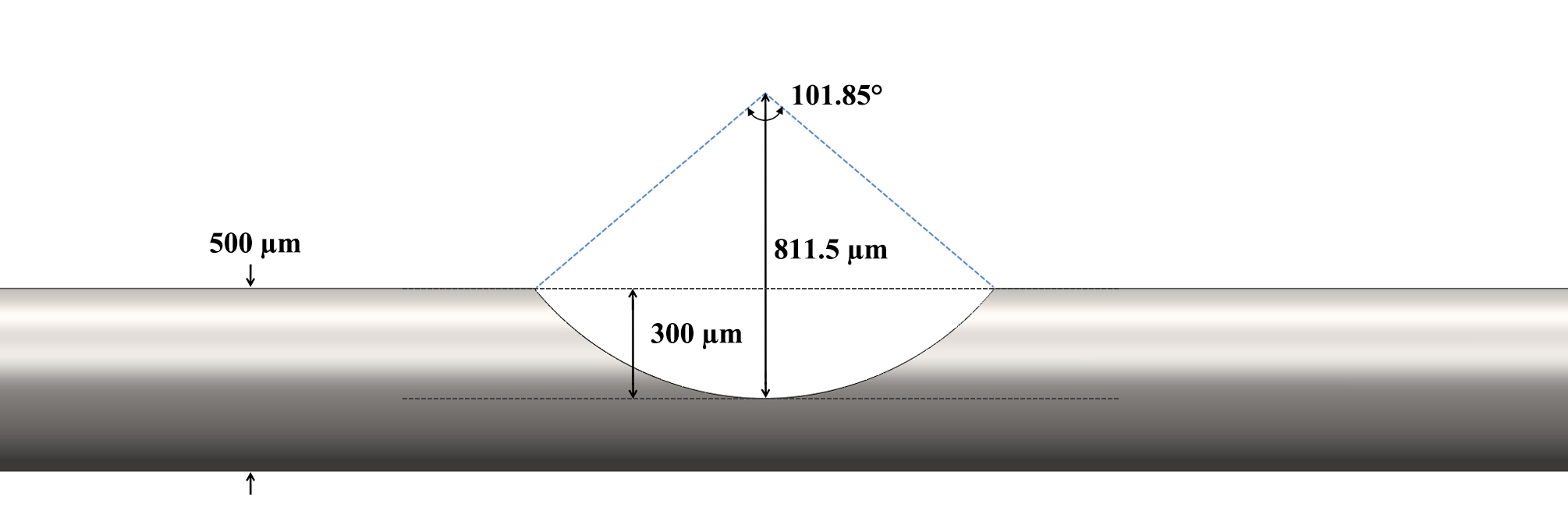

Supplement: S1 Fig — (JPG) [file pone.0210993.s001.jpg]

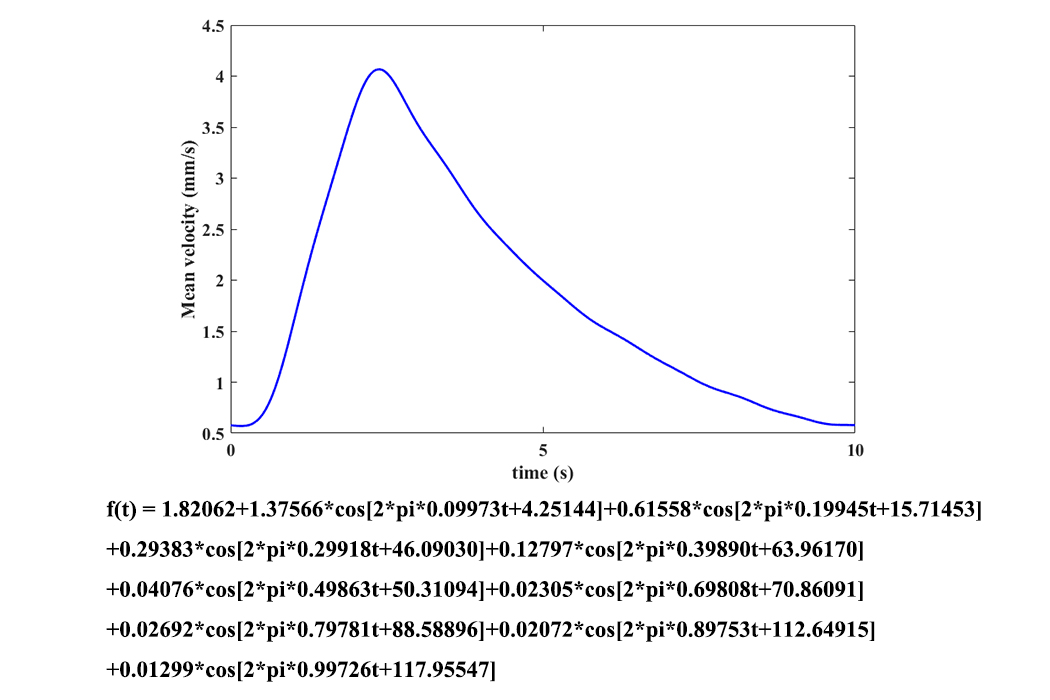

Supplement: S2 Fig — The mean velocity value is obtained by integrating 2D velocity profile of PBS for each time. (JPG) [file pone.0210993.s002.jpg]

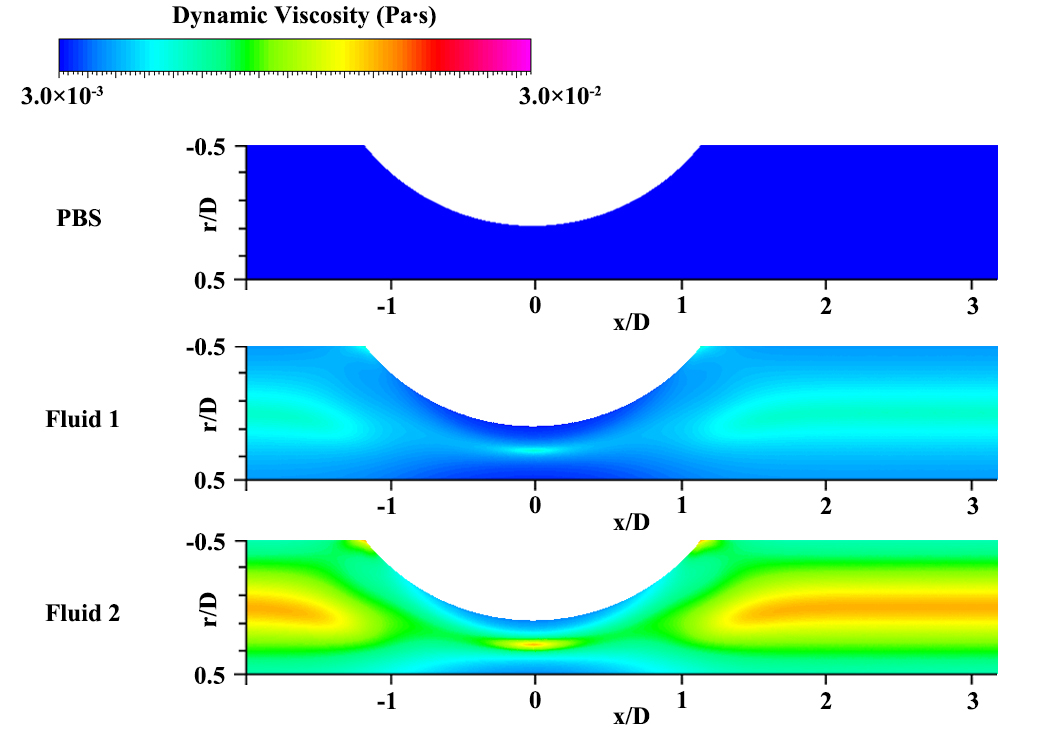

Supplement: S3 Fig — (JPG) [file pone.0210993.s003.jpg]
